# Mapping mammography in Arkansas: Locating areas with poor spatial access to breast cancer screening using optimization models and geographic information systems

**DOI:** 10.1017/cts.2020.28

**Published:** 2020-03-24

**Authors:** Sean G. Young, Meghan Ayers, Sharp F. Malak

**Affiliations:** 1 Department of Environmental and Occupational Health, University of Arkansas for Medical Sciences, Little Rock, AR, USA; 2 Department of Epidemiology, University of Arkansas for Medical Sciences, Little Rock, AR, USA; 3 Department of Radiology, University of Arkansas for Medical Sciences, Little Rock, AR, USA

**Keywords:** Breast cancer, mammography, screening, GIS, accessibility, rural health

## Abstract

**Introduction:**

Arkansans have some of the worst breast cancer mortality to incidence ratios in the United States (5th for Blacks, 4th for Whites, 7th overall). Screening mammography allows for early detection and significant reductions in mortality, yet not all women have access to these life-saving services. Utilization in Arkansas is well below the national average, and the number of FDA-approved screening facilities has decreased by 38% since 2001. Spatial accessibility plays an important role in whether women receive screenings.

**Methods:**

We use constrained optimization models within a geographic information system (GIS) to probabilistically allocate women to nearby screening facilities, accounting for facility capacity and patient travel time. We examine accessibility results by rurality derived from rural–urban commuting area (RUCA) codes.

**Results:**

Under most models, screening capacity is insufficient to meet theoretical demand given travel constraints. Approximately 80% of Arkansan women live within 30 minutes of a screening facility, most of which are located in urban and suburban areas. The majority of unallocated demand was in Small towns and Rural areas.

**Conclusions:**

Geographic disparities in screening mammography accessibility exist across Arkansas, but women living in Rural areas have particularly poor spatial access. Mobile mammography clinics can remove patient travel time constraints to help meet rural demand. More broadly, optimization models and GIS can be applied to many studies of healthcare accessibility in rural populations.

## Highlights

- Eighty percent of Arkansan women aged 40–84 years live within 30 minutes of a screening mammography facility.
- With travel time and capacity constraints, the recommended number of annual screenings cannot be provided by existing facilities.
- Small towns and Rural areas account for between 63% and 96% of unallocated demand.

## Introduction

Screening mammography (the use of x-ray imaging of the breast to check for breast cancer in women without signs or symptoms of disease) enables early detection and as much as a 40–67% reduction in breast cancer mortality [1]. Not all women have ready access to these services, with various socioeconomic, cultural, and geographic barriers leading to low utilization rates among certain populations [2,3]. In particular, health disparity populations of Blacks/African Americans, low-income populations, and rural populations tend to have low utilization rates [4–6]. Low screening utilization in turn translates into delayed diagnosis and decreased survival rates [7–11]. Curtis et al. found that differences in screening behaviors accounted for a considerable portion of mortality differences between populations [12]. Utilization in Arkansas is below the national average, with less than two-thirds of women aged 40 years and older reporting a mammogram in the past 2 years [13]. In addition, mortality to incidence ratios for breast cancer in Arkansas are among the worst in the United States (5^th^ for Blacks, 4^th^ for Whites, 7^th^ overall), and Black Arkansans have a 50% higher mortality rate for breast cancer than White Arkansans [14]. Screening mammography is likely the single most important modifiable behavior for reducing breast cancer mortality risk, with the potential to eliminate observed disparities in mortality.

Accessibility, measured using travel times between patients and clinics, has long been identified as an important determinant of healthcare utilization for breast cancer, particularly for rural populations [15,16]. DeSantis et al. suggest that racial and socioeconomic disparities with regard to stage at diagnosis and tumor size can be largely explained by disparities in access to screening services [17]. Nattinger et al. identified travel distance as inversely related to utilization of breast cancer treatment [18]. Meden et al. found travel distance was also associated with key treatment decisions among rural populations in Michigan, with those living farther away from clinics more likely to undergo radical mastectomies [19]. Simple models of accessibility use Euclidean (straight-line) distance between patients and facilities, assuming that everyone living within a specified distance of a facility have adequate access. These simple models ignore two important considerations: (1) patients travel along road networks, not in a straight-line and (2) facilities have limited capacity and cannot necessarily serve all patients within the specified travel distance [20]. Measures of *spatial accessibility* consider both access to care (the number of service locations within specific travel time thresholds) and availability of care (capacity or supply of services at accessible locations) [21].

Women living in Rural areas have particularly poor spatial access to screening due to the unequal distribution of screening facilities [5,22]. Gentil et al. found women in France living in Rural areas, economically deprived areas, or more than 30 minutes from a specialist breast cancer center were less likely to receive specialized care and had poorer survival prospects [23]. Spatial accessibility to screening facilities is likely to play an important role in whether or not women in Arkansas receive mammography screenings [24]. In a study using utilization data from 1997, Jazieh and Soora found that while over 50% of women in Arkansas self-reported screening, less than 23% actually received mammography screening [25]. Since that time the population in Arkansas has increased by 20% from 2.5 million to over 3 million people, and the number of FDA-certified mammography facilities in Arkansas has decreased by 38%, exacerbating disparities in spatial access, particularly for rural women. In fact, a recent study found Arkansas had the lowest spatial accessibility to mammography facilities of all states in the Lower Mississippi Delta Region [26].

Our objective is to map both the supply of and theoretical demand for screening mammography services in Arkansas, comparing demand scenarios according to different screening guidelines, and identify locations where demand cannot be met due to poor spatial access. Several national and international agencies and healthcare organizations provide guidelines for women regarding screening mammography use (see Table 1). It is not known to what extent the existing facilities that provide mammography services are able to meet demand, nor which areas of the state have the greatest unmet need. By using the road network distance to measure travel times instead of using Euclidean distance, we better capture real-world patient travel. By determining not only the number and location of mammography facilities providing screenings but also estimates of their screening capacity, we will obtain a more complete understanding of the true availability of mammography services in the state, allowing us to measure spatial accessibility. Intervention programs can use the resulting models for both planning and evaluation purposes.

**Table 1.** Description of theoretical demand scenarios for screening mammograms in Arkansas in 2017

Scenario	Agencies*	Operationalized Parameters	Theoretical Demand in 2017
1	ACOG, ACR, AMA, NCBC, NCCN, SBI	Annual screenings for ages 40–84	708,667
2	ACS, ASBS, ASCO	Annual screenings for ages 45–54; Biennial for ages 55–79	387,522
3	IARC	Annual screenings for ages 50–69	377,152
4	AAFP, ACP, USPSTF	Biennial screenings for ages 50–74	221,217

* AAFP – American Academy of Family Physicians; ACS – American Cancer Society; ACP – American College of Physicians; ACR – American College of Radiologists; ACOG – American Congress of Obstetricians and Gynecologists; AMA – American Medical Association; ASBS – American Society of Breast Surgeons; ASCO – American Society of Clinical Oncology; NCBC – National Consortium of Breast Centers; NCCN – National Comprehensive Cancer Network; SBI – Society of Breast Imaging; IARC – International Agency for Research on Cancer; USPSTF – US Preventive Services Task Force.

## Materials and Methods

Under the Mammography Quality Standards Act of 1992, the FDA certifies mammography facilities meeting baseline quality standards. Data on certified clinics, including street address and contact information, are available through the FDA’s Mammography Facility Database (https://www.accessdata.fda.gov/scripts/cdrh/cfdocs/cfMQSA/mqsa.cfm), updated weekly. To measure access in 2017, we used clinics listed as of January 2018 and geocoded to the street address level using ArcGIS 10.7 (Esri, Redlands, CA). Mobile clinics were excluded from the travel time analysis because their listed address in the database does not reflect the locations they serve. Instead we considered mobile clinics as universally accessible facilities subject only to capacity constraints. Data from the Arkansas Department of Health were used to determine the number of machines at each facility. Facilities were contacted to confirm street address and estimate screening capacity, and approximately 25% provided capacity estimates. Two facilities indicated that they no longer perform screening mammograms and were excluded from the analysis. For those facilities that could not be contacted or that were unable/unwilling to provide capacity estimates, facility capacity was calculated as three mammograms per machine per business hour, according to the 2006 Government Accountability Office definition of maximum capacity [27]. We further estimated approximately 75% of mammograms performed are screening mammograms [28], giving an estimated average of 4,500 screening mammograms per machine per year.

Data on the adult female population in Arkansas were obtained from the American Community Survey of the US Census. We used 5-year estimates for 2012–2017 at the Census Tract scale and mapped the distribution of women aged 40–84 years. In order to operationalize and compare different agencies’ screening guidelines, we made simplifying assumptions following the procedures outlined by Arleo et al [29]. If screening frequency was not specified, annual screening was assumed. For biennial recommendations, each woman in the relevant age range was counted as 0.5 to estimate annual demand. If screening was deemed optional or at patient’s request, no demand was added. If stopping age was described in terms of life expectancy, “<5–7 years” was set at age 84 and “<10 years” was set to 79. When operationalized to annual estimates for women of average risk, many of these agencies’ guidelines converged, resulting in four theoretical demand scenarios (see Table 1). Scenario 1 includes guidelines from six agencies and recommends annual screenings from ages 40 to 84. Scenario 2 includes guidelines from three agencies and recommends annual screenings for women aged 45–54 years, then biennial screenings from 55 to 79. Scenario 3 comes from a single agency and recommends annual screenings from ages 50 to 69. Scenario 4 includes guidelines from 3 agencies and recommends biennial screenings for ages 50–74.

To estimate road network travel times to screening facilities, we used Network Analyst in ArcGIS Pro (Esri, Redlands, CA) to create an origin–destination cost matrix between tract centroids and facility locations. We then created constrained optimization models with capacitated supply (number of screenings available at each facility) and apportioned demand (number of annual screenings required based on population and guideline-based theoretical demand scenarios) to probabilistically allocate theoretical demand to the nearest screening facilities with available capacity to minimize overall travel times [30,31]. Each model begins by allocating demand from a tract centroid to the nearest facility within the maximum travel time threshold until all demand in that tract is allocated or all capacity at the selected facility is exhausted. If demand remains unallocated, the next closest facility within the maximum travel time threshold is selected and the allocation continues. A new tract is then selected and its demand is allocated. This process is repeated until an end condition is met: (1) all demand is successfully allocated, (2) all capacity has been exhausted, or (3) no more demand can be allocated within travel time constraints. We compared optimization models for each demand scenario with different maximum travel time thresholds of 30 and 60 minutes. We also created models with no travel time threshold for comparison, to demonstrate the importance of travel time constraints for rural populations.

**Fig. 1.**
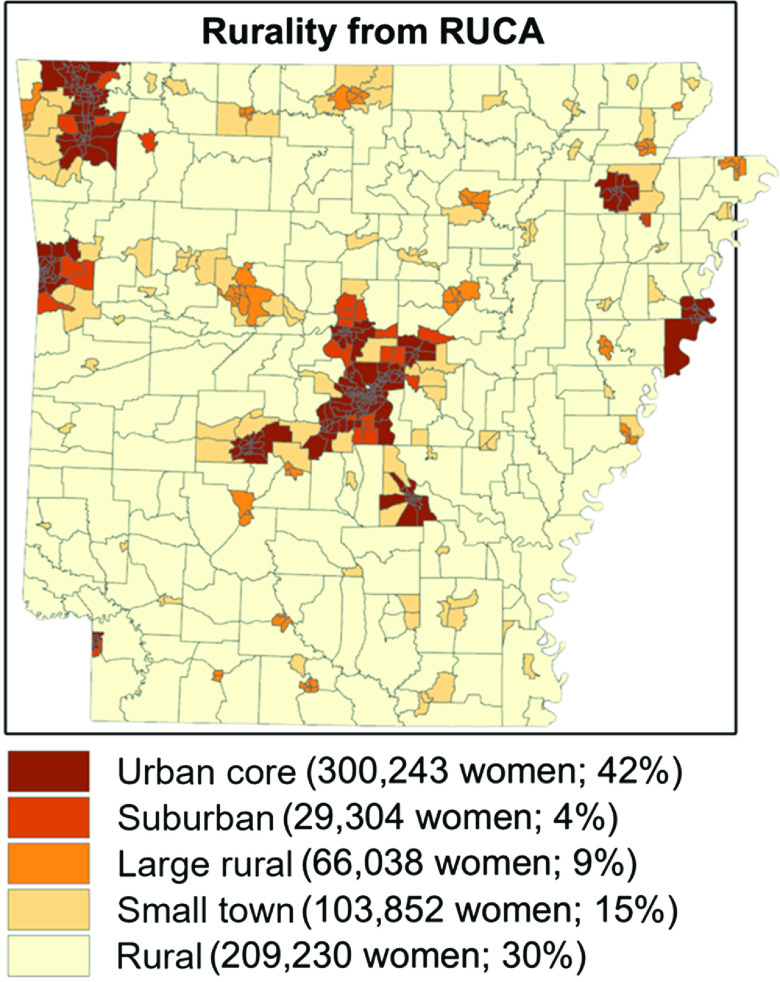
Rurality in Arkansas, derived from rural–urban commuting area (RUCA) codes, with the number of women aged 40–84 years in each category noted.

Rurality was evaluated using rural–urban commuting area (RUCA) codes [32,33], consolidated down to 5 levels of increasing rurality following Scheme 3 from the Washington State Department of Health Guidelines [34] (see Figure 1). These 5 categories are Urban core areas (RUCA code 1), Suburban areas (RUCA codes 2 and 3 with a population density of 100+ per square mile), Large Rural areas (RUCA codes 4–6 with a population density of 100+ per square mile), Small towns (RUCA codes 7–10 or any nonurban core area with population density between 50 and 100 per square mile), and Rural areas (including all locations outside the Urban core areas with a population density less than 50 per square mile). This classification scheme allows for areas with poor spatial accessibility to be examined and compared with regards to rurality at a higher resolution than traditional urban/rural dichotomies.

## Results

**Fig. 2.**
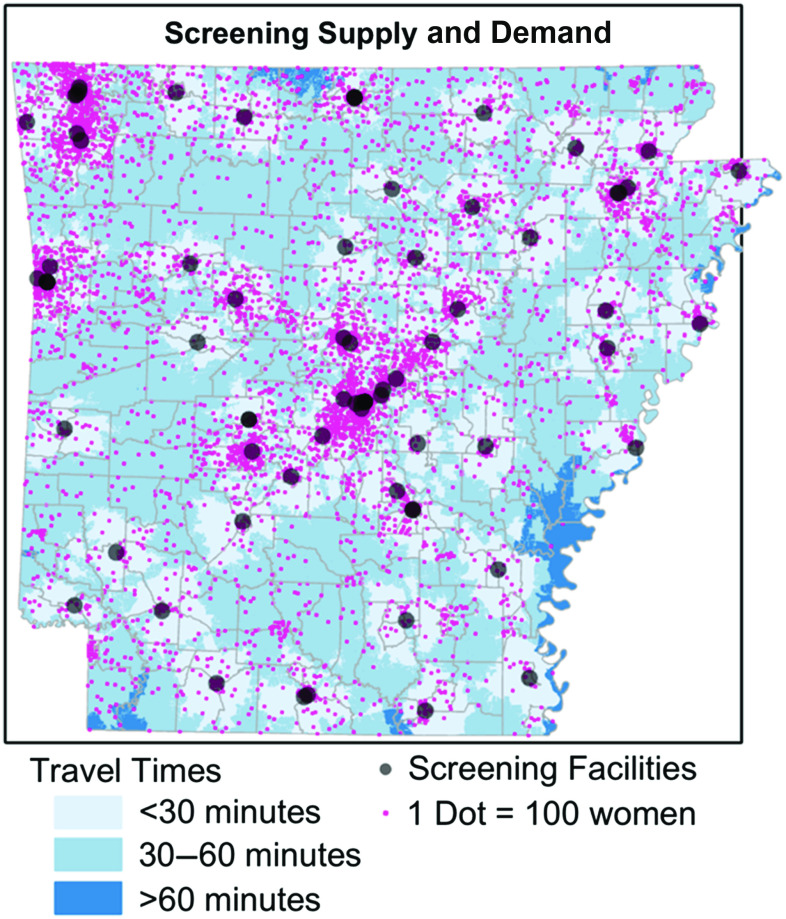
Distribution of women aged 40–84 years (1 pink dot equals 100 women), along with locations of current screening facilities (gray dots) and travel times to those facilities.

Total theoretical demand for annual screenings ranged from 708,667 to as few as 221,217 depending on the guideline scenario (see Table 1). The estimated total annual screening mammography capacity in the state (including approximately 10,000 screenings from mobile mammography units) was estimated at 419,000 screenings. Approximately 300,000 women aged 40–84 years (42%) live in Urban core areas, with another 95,000 (13%) living in Suburban and Large Rural areas. Approximately 104,000 women (15%) live in Small towns and 209,000 (30%) live in Rural areas. In contrast, more than half (56%) of screening facility capacity is located in Urban core areas, with Suburban and Large Rural areas contributing an additional 20% of total capacity. Small town and Rural areas contain only 24% of screening capacity. Approximately 502,300 women aged 40–84 years (80% of demand in Scenario 1) live within 30 minutes of a screening facility and nearly 100% live within 60 minutes of a facility. Locations with travel times greater than 1 hour from the nearest screening facility were sparsely populated. Figure 2 shows the distribution of screening facilities, the distribution of women aged 40–84 years (each pink dot represents 100 women), and travel time polygons for each facility.

In all 4 scenarios, theoretical demand could not be completely allocated to screening facilities within the maximum travel time thresholds of 30 or 60 minutes. Using a 30-minute threshold, between 68,944 and 339,120 women were unable to be allocated to an existing facility. Using a 60-minute threshold, between 3,641 and 291,112 women were unable to be allocated. Removing all travel time constraints, total capacity was still insufficient to meet theoretical demand in Scenario 1 (289,667 women unallocated), but was sufficient for Scenarios 2–4. Unallocated demand for each scenario (after adjusting for contributions from mobile mammography units) is indicated in Table 2, stratified by rurality.

**Table 2.** Unallocated theoretical demand for screening mammograms (i.e. the number of mammograms needed to meet scenario guidelines that could not be supplied), stratified by demand scenario, maximum travel time threshold, and rurality. Note that totals are adjusted to reflect the contributions of mobile mammography clinics, while values stratified by rurality are not

	Unallocated Theoretical Demand			
	Scenario 1	Scenario 2	Scenario 3	Scenario 4
30 Minutes	339,120	142,031	138,263	68,944
Urban core	90,125	23,203	22,148	8,653
Suburban	18,767	7,340	7,180	3,296
Large rural	16,419	4,866	4,671	1,824
Small town	54,511	26,751	24,956	13,282
Rural	169,298	89,872	89,308	51,891
60 Minutes	291,112	28,597	27,540	3,641
Urban core	80,671	3,426	2,562	329
Suburban	15,551	335	368	0
Large rural	16,043	1,951	1,848	248
Small town	47,284	5,157	4,905	1,372
Rural	141,563	27,728	27,857	11,693

**Fig. 3.**
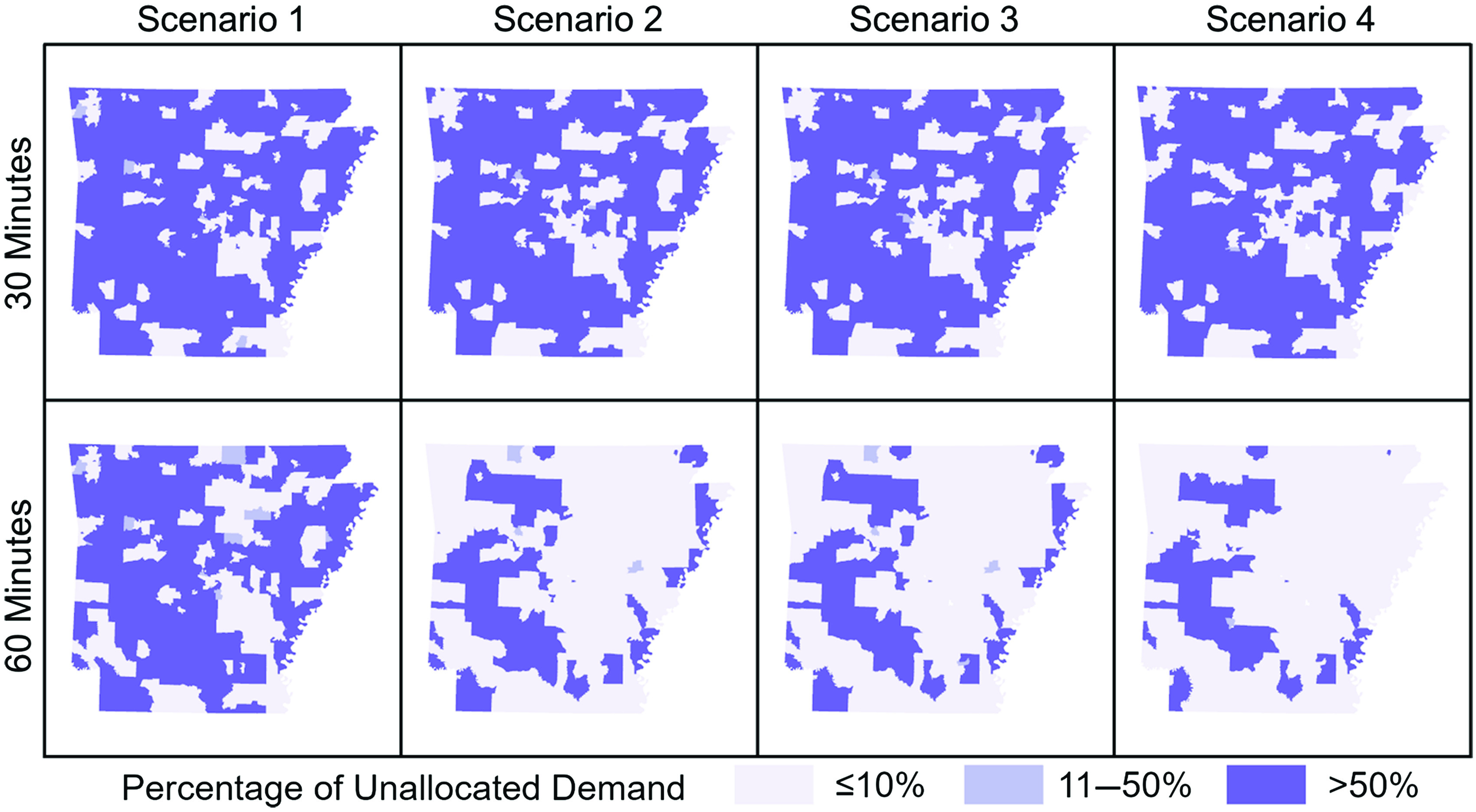
Percentage of unallocated theoretical demand for screenings by scenario and travel time threshold. Darker shades indicate a higher proportion of women living in that area lack spatial access to screening.

Unallocated demand was not evenly distributed across the state, with Small town and Rural areas accounting for between 63% and 96% of total unallocated demand in all models except those lacking travel time thresholds. When increasing the travel time threshold from 30- to 60 minutes, in each scenario the greatest absolute reduction in unallocated demand was in Rural areas. As the total theoretical demand decreased from Scenarios 1 to 4, the proportion of unallocated demand in Urban core and Suburban areas also decreased from Scenarios 1 to 4, while the proportion of unallocated demand located in Rural areas increased. With a 60-minute threshold, Scenario 1 allocated an additional 48,000 women beyond the 30-minute threshold model, over half (58%) of which were from Rural areas. Geographic distributions of unallocated demand in all models are shown in Figure 3. The geographic pattern is largely consistent between all demand scenarios under a 30-minute maximum travel time constraint. With a 60-minute threshold the patterns diverge with Scenarios 2–4 showing substantially more areas with 90% or more spatial access. When no travel time thresholds were employed, only Scenario 1 presents any regional disparities while all theoretical demand was allocated in Scenarios 2–4. In all models, Urban core areas and most Suburban areas of the state were categorized as the most allocated areas, with less than or equal to 10% of the screening demand in those areas being unallocated to a facility. In contrast, Rural areas were much more likely to be found in the least allocated category, with more than half of the screening demand remaining unallocated in most models. For comparison, we also performed the analysis using Zip Code Tabulation Areas, which demonstrated the same trends and patterns (see Supplemental Table 1).

## Discussion

Geographic disparities in spatial access to screening mammography exist across Arkansas, but are most pronounced in Small towns and Rural areas. In all models, the largest proportion of unallocated demand was located in Rural areas. This is not surprising considering more women aged 40–84 years live in Small town and Rural areas than in Urban core areas in Arkansas. Furthermore, Scenarios 2–4 resulted in higher proportions of unallocated demand being located in Rural areas compared to Scenario 1. Given travel constraints, current screening facilities in Arkansas have insufficient capacity to meet theoretical demand, even in many regions within 30 minutes of facilities. Such facilities may be accessible geographically, but they are not universally available due to capacity constraints. Only when travel time constraints are removed can all theoretical demand be met in Scenarios 2–4; however, theoretical demand in Scenario 1 cannot be met even under these conditions. Policy makers may be interested to see how different screening guidelines influence spatial accessibility results by rurality.

This research was subject to important limitations. First, a lack of information regarding locations visited by mobile clinics in the state prohibited us from including them in the optimization models. Our inclusion of their screening capacity in state totals allowed us to estimate their overall contribution, but we were unable to determine their relative impact by rurality. We also made simplifying assumptions regarding travel behavior, namely that patients will visit the nearest available facility based on distance from their place of residence and travel via personal vehicle. Alford-Teaster et al. examined mammography utilization among 646,553 women in the United States and found 35% of women used the closest facility, and of those that did not, 75% used a facility within 5 minutes of the closest facility, indicating the closest facility assumption is a reasonable approximation for the majority of women in the United States [35]. Individual-level information would be needed to make notable improvements in travel time estimates, although estimates from the US Census of the percentage of households without vehicles could be used to parameterize a multimodal optimization model that includes travel time via walking and/or public transit [20]. Another challenge was the low response rate (25%) from clinics regarding screening capacity. Previous survey efforts among screening facilities in the state have met with similarly low response rates (26% in 2016, unpublished data). Many were unwilling to share information, possibly for perceived competitive reasons.

Access to care is a multidimensional concept including availability of services (capacity), accessibility (travel constraints), affordability, and acceptability in terms of patient preferences, among others [36,37]. Our optimization models of spatial accessibility only considered the first two components, availability and accessibility; however, optimization models are customizable and can be expanded to incorporate nonspatial considerations [30]. We anticipate future modeling efforts in this area will include such advancements. Of particular interest is the role of insurance status on accessibility and utilization [17]. The Affordable Care Act expanded access to prevention coverage for women’s health and well-being. It required that screening mammography must be covered and that plans can no longer charge a patient a copayment, coinsurance, or deductible for this service when they are delivered by a network provider. The Health Resources and Services Administration states “Screening for breast cancer by mammography in average-risk women no earlier than age 40 and no later than age 50. Screening should continue through at least age 74 and age alone should not be the basis to discontinue screening. Screening mammography should occur at least biennially and as frequently as annually” (https://www.hrsa.gov/womens-guidelines/index.html).

Public health interventions seeking to improve access, including educational campaigns and mobile mammography clinics (which bring screening mammography equipment and trained personnel to regions without existing facilities), can use these results to target those particular rural communities in the state most likely to experience disparities based on poor spatial accessibility. More broadly, optimization models can be used to evaluate spatial accessibility to a wide range of healthcare services. Furthermore, these models can be used to predict the impact of proposed interventions. For example, if there were plans to build a new screening clinic in a rural community, planners could add the clinic to these models to determine the probable impact on spatial accessibility within the state, or could even compare several potential locations to choose the clinic site that maximizes spatial accessibility. Similarly, new and/or existing mobile clinics could use these models to determine which rural communities to visit by identifying those with the poorest current spatial access – indeed, this was one of the primary motivating factors for the creation of these models. While it may not be possible to remove all barriers to access, mobile mammography clinics have the ability to effectively eliminate (or at least minimize) travel time barriers from patients by bringing screening services to their communities. A related issue is spatial access to follow-up services including diagnostic imaging, biopsies, and cancer treatment services. The models described herein are also appropriate for these subsequent analyses. Increasing spatial access to these life-saving services in rural communities is the first step toward reducing breast cancer mortality disparities in Arkansas and beyond.

## Supplementary material

For supplementary material accompanying this paper visit https://doi.org/10.1017/cts.2020.28. click here to view supplementary material
