# Supplementary material for: Mapping mammography in Arkansas: Locating areas with poor spatial access to breast cancer screening using optimization models and geographic information systems
Source: J Clin Transl Sci. 2020 Mar 24;4(5):437–42. doi: 10.1017/cts.2020.28 (PMC7681135; doi:10.1017/cts.2020.28)
Supplement: Supplementary file 1 [file S205986612000028Xsup001.docx]

Supplemental Materials

Supplemental Table 1 Unallocated theoretical demand for screening mammograms (i.e. the number of mammograms needed to meet scenario guidelines that could not be supplied) at the Zip Code Tabulation Area scale, stratified by demand Scenario, maximum travel time threshold, and rurality. Note that totals are adjusted to reflect the contributions of mobile mammography clinics, while values stratified by rurality are not.

|  |  | Unallocated Theoretical Demand | | | |
| --- | --- | --- | --- | --- | --- |
|  |  | Scenario 1 | Scenario 2 | Scenario 3 | Scenario 4 |
| 30 Minutes | | 361,221 | 141,866 | 138,612 | 75,495 |
|  | Urban core | 95,423 | 24,820 | 23,815 | 10,948 |
|  | Suburban | 25,380 | 3,774 | 3,369 | 1,992 |
|  | Large rural | 17,001 | 4,317 | 3,665 | 1,765 |
|  | Small town | 55,294 | 22,856 | 21,964 | 11,024 |
|  | Rural | 16,1036 | 82,308 | 82,101 | 47,562 |
| 60 Minutes | | 305,428 | 40,926 | 39,528 | 12,848 |
|  | Urban core | 81,804 | 6,151 | 5,657 | 446 |
|  | Suburban | 22,364 | 510 | 12 | 6 |
|  | Large rural | 14,948 | 960 | 632 | 0 |
|  | Small town | 47,927 | 4,083 | 3,886 | 351 |
|  | Rural | 131,298 | 29,223 | 29,341 | 12,045 |
